# A Multidimensional Computerized Adaptive Short-Form Quality of Life Questionnaire Developed and Validated for Multiple Sclerosis: The MusiQoL-MCAT

**DOI:** 10.1097/MD.0000000000003068

**Published:** 2016-04-08

**Authors:** Pierre Michel, Karine Baumstarck, Badih Ghattas, Jean Pelletier, Anderson Loundou, Mohamed Boucekine, Pascal Auquier, Laurent Boyer

**Affiliations:** From the Aix-Marseille University, EA 3279 – Public Health, Chronic Diseases and Quality of Life – Research Unit (PM, KB, BG, AL, MB, PA, LB); Aix-Marseille University – I2 M UMR 7373 – Mathematics Institute of Marseille (PM, BG); and Departments of Neurology and CRMBM CNRS6612, La Timone University Hospital, APHM, Marseille, France (JP).

## Abstract

The aim was to develop a multidimensional computerized adaptive short-form questionnaire, the MusiQoL-MCAT, from a fixed-length QoL questionnaire for multiple sclerosis.

A total of 1992 patients were enrolled in this international cross-sectional study. The development of the MusiQoL-MCAT was based on the assessment of between-items MIRT model fit followed by real-data simulations. The MCAT algorithm was based on Bayesian maximum a posteriori estimation of latent traits and Kullback–Leibler information item selection. We examined several simulations based on a fixed number of items. Accuracy was assessed using correlations (*r*) between initial IRT scores and MCAT scores. Precision was assessed using the standard error measurement (SEM) and the root mean square error (RMSE).

The multidimensional graded response model was used to estimate item parameters and IRT scores. Among the MCAT simulations, the 16-item version of the MusiQoL-MCAT was selected because the accuracy and precision became stable with 16 items with satisfactory levels (*r* ≥ 0.9, SEM ≤ 0.55, and RMSE ≤ 0.3). External validity of the MusiQoL-MCAT was satisfactory.

The MusiQoL-MCAT presents satisfactory properties and can individually tailor QoL assessment to each patient, making it less burdensome to patients and better adapted for use in clinical practice.

## Abstract 2

Supplemental Digital Content is available in the text

## INTRODUCTION

Health-related quality of life (QoL) measurements are increasingly being considered important in regard to evaluating disease progression, treatment options, and the management of care provided to patients with chronic diseases.^1,2^ Self-reported questionnaires are traditionally used to measure QoL, but they are often considered too lengthy by patients and professionals.^3^ The time and resources necessary for the completion of questionnaires are constraints on professionals whose main role is providing patient care.^4^ Additionally, questionnaires should be as brief as possible because of the difficulties of fatigue and concentration in some clinical populations (e.g., patients with multiple sclerosis [MS], schizophrenia). Providing shorter questionnaires in QoL measures may be useful for clinical practice.^5^ Short-form instruments are usually a fixed-length (i.e., the same items are proposed to all patients) and adapted from a long-form instrument by reducing the number of questions based on classical and item response theories (IRTs). However, these fixed-length short-form instruments have drawbacks (e.g., the reduction of questions brings a risk of losing important information that can result in a decline of measurement precision).^6,7^ Additionally, because some items are not tailored to patients, the precision of the QoL measure is not maximized, and patients may feel a lack of interest in the QoL measure and stop completing the questionnaire.

Interestingly, methods based on IRT models, currently used in the development of unidimensional item banks and computerized adaptive testing (CAT), can be adapted to overcome the problems faced by the development of fixed-length short-form questionnaires.^8,9^ Indeed, CAT allows for the administration of only the items that will offer the most relevance for a given individual, reducing the length of the questionnaire and the completion time in addition to maintaining the test's precision.^10–12^ Additionally, multidimensional CAT (MCAT) based on multidimensional IRT (MIRT) has been recently applied to measure health problems in various chronic diseases (e.g., symptomatology, fatigue, physical, and emotional functioning).^13–18^ Because of the multidimensional nature of QoL, this method seems relevant in developing a valid and reliable adaptive short-form QoL questionnaire.^14^ Currently, MCATs applied to shorten fixed-length available QoL questionnaires are scarce.^14,19^

The aim of this study was to develop a multidimensional computerized adaptive short-form questionnaire (MCAT) from a fixed-length available QoL questionnaire for patients with a chronic disease marked by the difficulties of fatigue and concentration, MS. Our study focused on the multiple sclerosis international quality of life questionnaire (MusiQoL), which is a widely used QoL questionnaire in MS.^20^ Compared to other MS questionnaires, this instrument has 3 important characteristics: specifically reflecting the perspective of patients with MS on the impact of the disease on their daily life; anchored in an explicit conceptual approach;^21^ and developed and available in multiple languages and psychometrically validated to appropriate standards.

## METHODS

### Questionnaire

The MusiQoL questionnaire is a MS-specific, self-administered, and multidimensional QoL instrument.^20^ It comprises 31 items describing 9 dimensions. Each dimension is named according to its constitutive items as follows: activities of daily living (ADL, 8 items), psychological well-being (PWB, 4 items), symptoms (SYMP, 4 items), relationships with friends (RFR, 3 items), relationships with family (RFA, 3 items), relationships with healthcare system (RHCS, i.e., satisfaction with healthcare; 3 items), sentimental and sexual life (SSL, 2 items), coping (COP, 2 items), and rejection (REJ, 2 items). Each item is scored on a 6-point Likert scale, in which a score of 1 represents never/not at all, 2 represents rarely/a little, 3 represents sometimes/somewhat, 4 represents often/a lot, 5 represents always/very much, and 6 represents not applicable. For each individual, the score on each dimension is obtained by computing the mean of the item scores for that dimension. All dimension scores are linearly transformed to a 0 to 100 scale. A global index score is computed as the mean of the dimension scores. Higher scores indicate a higher level of QoL.

### Study Design and Setting

Data from an international, multicenter, and cross-sectional MusiQoL validation study were used.^20^ Patients were recruited between January 2004 and February 2005 at neurological departments in 15 countries: Argentina, Canada, France, Germany, Greece, Israel, Italy, Lebanon, Norway, Russia, South Africa, Spain, Turkey, UK, and USA. This study was performed in accordance with the Declaration of Helsinki and all applicable regulatory authority requirements and national laws (Institutional Review Boards or Independent Ethics Committees in accordance with the local requirements of each of the 15 countries). Written informed consent from patients was obtained before any study procedures were performed.

### Population

The inclusion criteria included a diagnosis of MS according to McDonald,^22^ being treated as an in- or outpatient at a hospital, over 18 years of age, informed consent to participate, and a native speaker of the local language. The main exclusion criteria included a neurologic diagnosis other than MS, dementia, ongoing severe relapse, an inability to complete the questionnaire unassisted, and withdrawal of consent.

### Data Collection

In addition to the MusiQoL questionnaire, the following data were collected:

1. Socio-demographic information: age (years); gender (male, female); educational level (less than 12 years, greater than 12 years); marital status (single, not single); and employment status (active, unemployed).
2. Clinical data: disease duration (years); MS subtype (relapsing–remitting [RR], primary progressive [PP], secondary progressive [SP], and clinically isolated syndrome [CIS]);^23^ and MS disability using the expanded disability status scale (EDSS)^24^ (an ordinal clinical rating scale ranging from 0 [normal neurologic examination] to 10 [death due to MS]).
3. QoL was assessed using the SF-36,^25^ a generic questionnaire describing 8 subscales: physical function, social function, role-physical (RP), role-emotional (RE), mental health (MH), vitality, bodily pain, and general health. Two composite scores (physical and mental composite scores [PCS-SF-36] and [MCS-SF-36]) were calculated. The SF-36 yields scores on a 0 to 100 scale, in which 0 represents the lowest and 100 the highest QoL scores.

### MCAT Procedure and Analyses

This procedure was divided into 3 phases: MIRT analysis; MCAT simulations with analyses of accuracy and precision; and clinical validity of the MusiQoL-MCAT.

#### Multidimensional Item Response Theory Analysis

Percentages of missing values were computed for each item. In accordance with the steps taken previously to validate the MusiQoL,^20^ a between-items MIRT model was calibrated. We tested 2 flexible IRT models that allow for the consideration of items with various numbers of categories and various difficulty thresholds: the multidimensional graded response model (MRGM)^26^ and the multidimensional generalized partial credit model.^27^ The MRGM was retained because it yielded a better fit than multidimensional generalized partial credit model in regard to the Akaike's information criterion and Bayesian information criterion. We also tested 2 IRT models with missing data and imputed data. For the model with imputed data, we used multiple data imputation because we considered the data missing not at random, following previous works on QoL.^28–30^ The model with missing data was retained because it yielded a better fit in terms of the Akaike's information criterion (145,922 vs 153,334) and Bayesian information criterion (146,974 vs 154,359).

Item parameters were thus estimated using the MRGM with unconditional maximum likelihood (ML) estimation, as implemented in the R package *mirt*.^31^ We used the Metropolis–Hastings Robbins–Monro^32^ method as an estimation algorithm because it provides better precision than a classical expectation-maximization algorithm approach^33^ in the presence of more than 3 factors.

The MRGM consists of 2 multidimensional sequential 2-parameter logistic models and is defined as follows: 
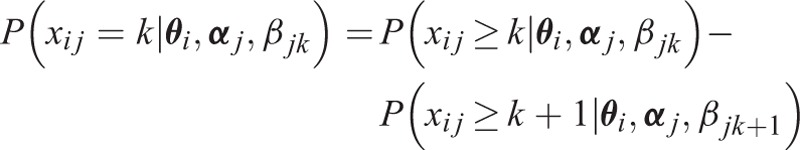


where 
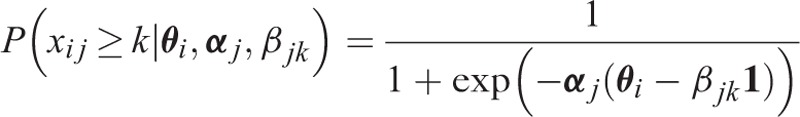


where i is the ith individual, j the jth item, x_ij_ the ordinal response taking the value  
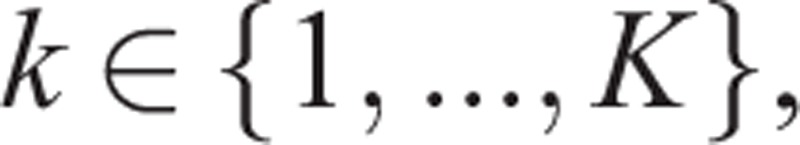
, α_j_ the item discrimination parameter according to dimension d, θ_i_ the individual parameter according to dimension *d*, and β_jk_ is the kth item difficulty threshold parameter.

Bayesian maximum a posteriori (MAP) estimation^8^ of person-specific parameters (i.e., latent trait estimates) were computed using the MRGM parameters and the 31 item responses, providing IRT dimension scores for each patient. In IRT, item information is a function of the item parameters (i.e., the discrimination and difficulty threshold parameters). An item with more information is more discriminant and provides a lower error of measurement. The test information is the sum of all item information. The contribution of each item to the total test information (also called the amount of test information) was calculated.

The unidimensionality of each dimension was assessed using a Rasch analysis. The goodness-of-fit statistics (inlier-sensitive fit, ranging between 0.7 and 1.3) ensured that all items of the scale measured the same concept.^34^

Differential item functioning (DIF) analyses were performed to compare the item differences among countries to determine whether all items behaved the same way.^35^ The DIF indicates whether an item performs and measures differently for 1 subgroup of a population compared with another.

#### MCAT Simulations With Analyses of Accuracy and Precision

We performed a post-hoc or real-data simulation approach (i.e., complete response patterns to the 31 items of the MuSiQoL were used to simulate the conditions of an MCAT assessment). The algorithm of the MCAT was based on Mulder and van der Linden's work for Kullback–Leibler Information Item Selection.^36^ Initially, the person-specific parameter estimate was set to the IRT dimension population mean scores. As the starting item, we used the item with the highest amount of test information. Item selection depended on responses to earlier items in the questionnaire taken from the empirical data. At each step of item selection, the Bayesian MAP procedure estimated the latent trait level that maximized the posterior distribution based on the current likelihood of the data and the assumed prior distribution. As a stopping criterion, we examined the 4 initial simulations based on a fixed number of items (5, 10, 15, and 20).

For each simulation, MCAT dimension scores were calculated, and accuracy and precision were then assessed. Accuracy was assessed using the level of correlation between the MCAT and the IRT dimension scores based on the full set of items (levels of correlation >0.9 were expected for each dimension). Precision was assessed using 2 indicators: the standard error measurement (SEM) and the root mean square error (RMSE). The SEMs of the MCAT dimension scores are considered indicators of reliability. The SEMs of the MCAT dimension scores are considered indicators of reliability. According to Harvill's work,^37^ there is a direct relationship between the reliability of a dimension and the SEM; lower reliability estimates provide higher SEM estimates. An acceptable range was defined as <0.55 to ensure a satisfactory reliability level (reliability >0.70). The RMSE shows how precise the MCAT dimension scores are relative to the IRT scores from the full item set. The RMSE is calculated as follows: 
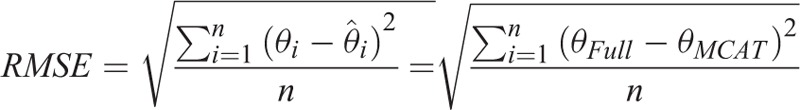


where *θ*_*i*_ is the IRT score from the full item set of the *i*th individual and  
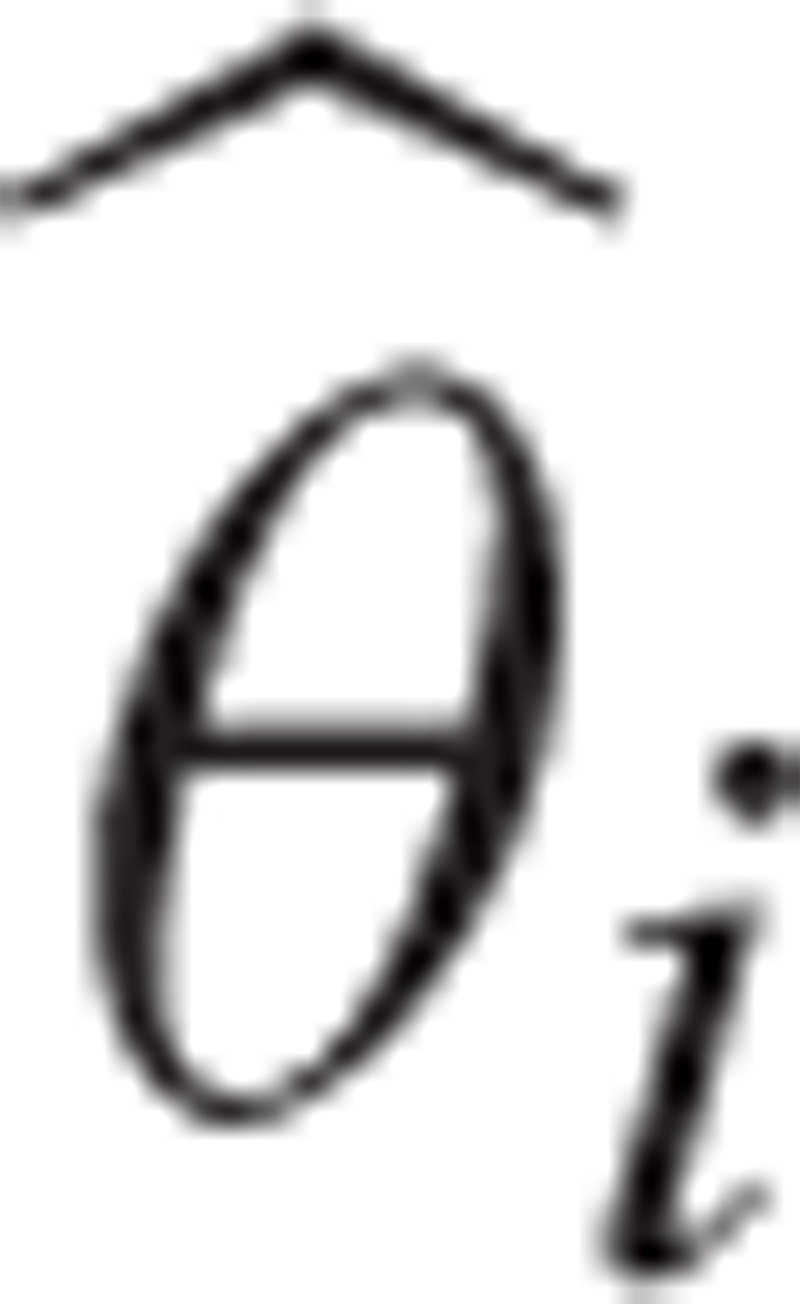
 is the MCAT score, and smaller values of RMSE represent better measurement precision. RMSE values lower or equal to 0.3 indicate excellent measurement precision.^38^

According to the accuracy/precision of the first 4 simulations, other simulations were tested to determine the best MCAT version. The final version of the MusiQoL-MCAT was selected considering the lowest number of items matching with the most satisfactory level of accuracy and precision. The item exposure (i.e., the number of times each item was exposed during the CAT procedure) was described for this version.

#### Validity of the MusiQoL-MCAT

To assess the validity of the selected MusiQoL-MCAT, we explored both convergent and discriminant validity. To explore the convergent validity, Pearson correlation coefficients were used to investigate the relationships between the dimensions of the MusiQoL-MCAT and the dimensions of the generic QoL questionnaire (i.e., SF-36). In accordance with the assumptions from the initial validation of the MusiQoL,^20^ we hypothesized that the MusiQoL-MCAT scores would be more correlated with scores of dimensions exploring similar aspects from the SF-36 than with those exploring dissimilar aspects. The discriminant validity was determined by exploring the relationships between the MusiQoL-MCAT scores and socio-demographic (i.e., age, gender, educational level, marital status, and employment status) and clinical (i.e., EDSS score and MS subtypes) features using *t*-tests, ANOVAs, and Pearson correlations. To control the familywise error rates caused by the large number of correlations, we performed multivariate permutation tests.^39,40^

Several hypotheses were formulated in accordance with previous studies: the MusiQoL-MCAT should differ according to sociodemographic characteristics (i.e., with younger age, higher educational level, and being in a couple associated with higher QoL); should be negatively correlated with the severity of the disease (i.e., EDSS); and should be lower in patients with the SP form of MS.

All the statistical analyses were performed using R version 2.15.2.

## RESULTS

The international field study sample included 1992 patients with MS. Patients were recruited from the 15 following countries: Argentina (n = 27), Canada (n = 77), France (n = 179), Germany (n = 209), Greece (n = 92), Israel (n = 66), Italy (n = 379), Lebanon (n = 20), Norway (n = 104), Russia (n = 201), South Africa (n = 53), Spain (n = 224), Turkey (n = 228), UK (n = 36), and USA (n = 97). The mean age was 42.2 (standard deviation, SD = 11.9) years; 1382 patients (70.5%) were female, and 578 patients (29.5%) were male; 592 (35.2%) had a high educational level; and 372 (21.7%) were single. Patients had an RR MS subtype in 70.4% of cases, SP in 21.0%, PP in 7.1%, and CIS in 1.5%. The median EDSS score was 3.0 (interquartile range = 3.5).

### Multidimensional Item Response Theory Analysis

**FIGURE 1:**
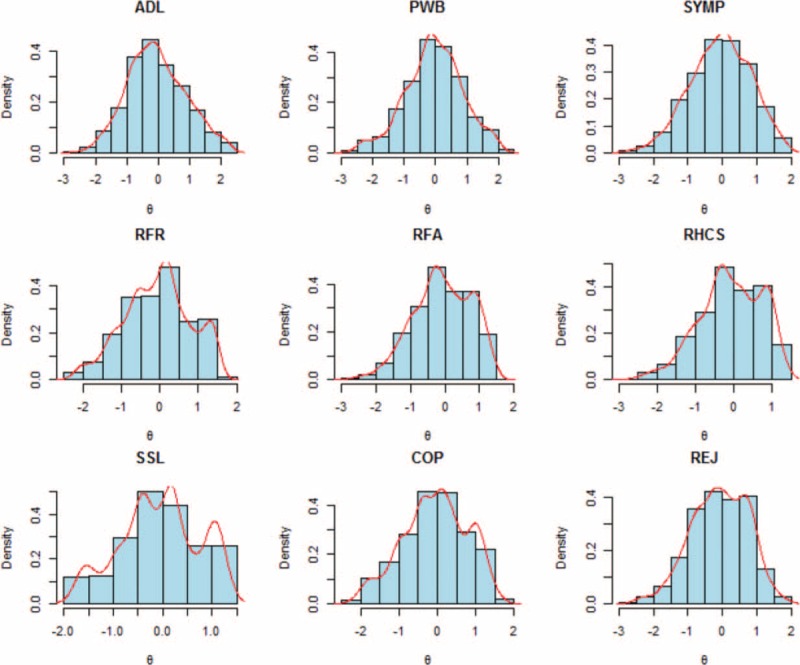
IRT score distribution for each MusiQoL dimension. ADL = activities of daily living, COP = coping, IRT = item response theory, MusiQoL = multiple sclerosis international quality of life questionnaire, PWB = psychological well-being, REJ = rejection, RFA = relationships with family, RFR = relationships with friends, RHCS = relationships with healthcare system, SSL = sentimental and sexual life, SYMP = symptoms.

Percentages of missing data, estimated item parameters, information, and inlier-sensitive fit are presented in Table 1, and the IRT score distribution for each dimension is presented in Figure 1. Item 17 from the RFR dimension (“have you felt understood by your friends?”) provided the greatest amount of information, and item 16 from the SYMP dimension (“have you experienced unpleasant feelings: i.e., hot, cold?”) provided the least amount of information. Substantial DIF between countries was not evidenced for all dimensions, confirming the interest of this MCAT in international studies.

**TABLE 1:**
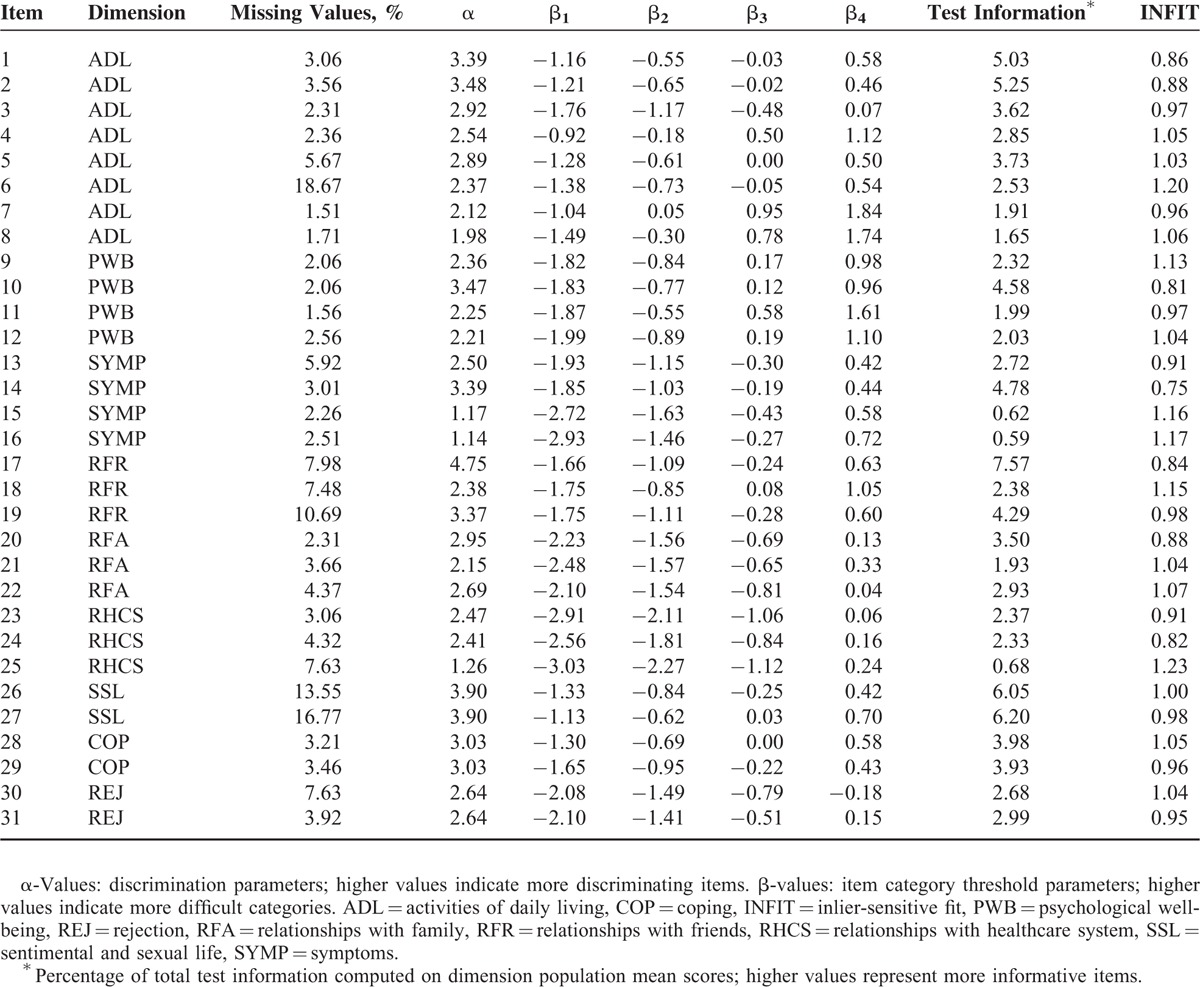
Estimated Item Parameters and Information

### Analyses of Accuracy and Precision

Real-data simulations were performed on 922 patients with complete response patterns to the 31 items of the MuSiQoL. Accuracy and precision indicators of each simulation are presented in Table 2.

**TABLE 2:**
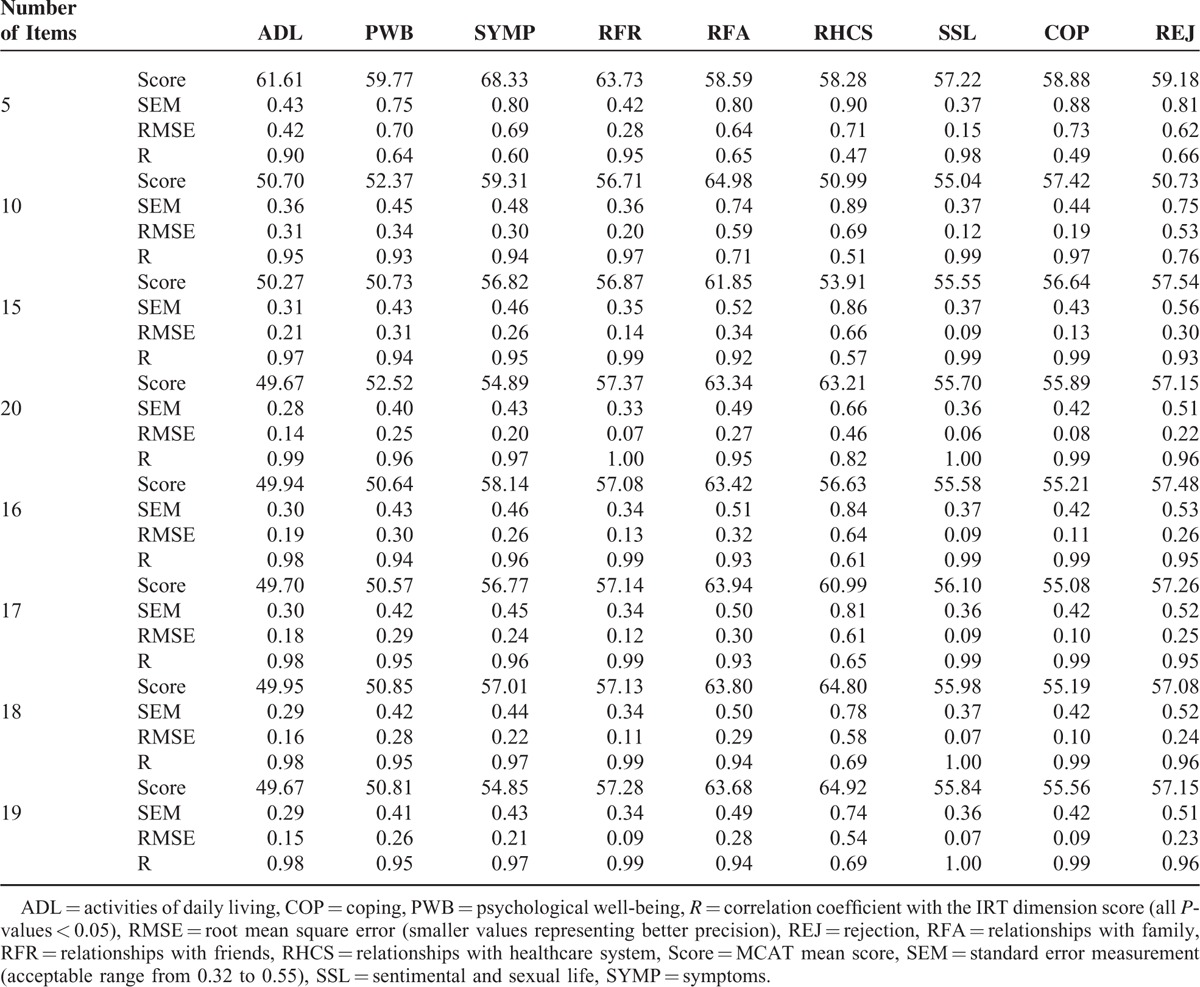
MCAT Simulations: Accuracy and Precision Parameters for Each Dimension

The number of dimensions with satisfactory accuracy (i.e., correlation >0.9) increased when simulations included a high number of items (from 3 of the 9 dimensions for the 5-item simulation to 8 of the 9 dimensions for the 20-item simulation). The relationships with healthcare system dimensions remained unsatisfactory regardless of the number of items in the simulation.

In regard to accuracy, the 2 indicators of precision were better when simulations included a high number of items. The number of dimensions with satisfactory SEM and RMSE varied from 3 of the 9 dimensions for the 5-item simulation to 8 of the 9 dimensions for the 20-item simulation and from 2 of the 9 dimensions for the 5-item simulation to 8 of the 9 dimensions for the 20-item simulation, respectively. The same dimension (i.e., relationships with the healthcare system) remained unsatisfactory regardless of the number of items in the simulation.

As accuracy and precision of the 15- and 20-item simulations were the most satisfactory, 4 supplementary simulations were tested from 16 to 19 items. The 16-item version of the MusiQoL-MCAT was defined as the most satisfactory MCAT simulation because the level of accuracy and precision did not substantially change after 16 items.

**FIGURE 2:**
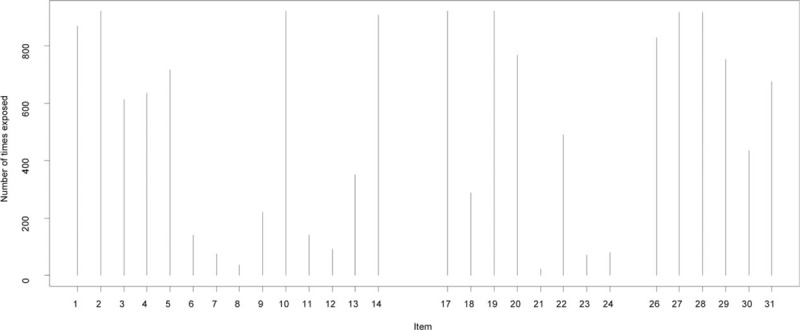
Item exposure for the selected computerized adaptive testing (CAT) procedure. Items: 1–8: activity of daily living; 9–12: psychological well-being; 13–16: symptoms; 17–19: relationships with friends; 20–22: relationships with family; 23–25: relationships with health care system; 26, 27: sentimental and sexual life; 28, 29: coping; 30–31: rejection.

Item exposure (i.e., the utilization frequency of an item) of the 16 item version of the MusiQoL-MCAT procedure is presented in Figure 2. Three items from both the SYMP and RHCS dimensions were never administered (items 15, 16, and 25), whereas 8 were administered more than 9 times out of 10 (items 1 and 2 from ADL dimension, item 10 from PWB dimension, item 14 from SYMP dimension, items 17 and 19 from RFR dimension, item 27 from SSL dimension, and item 28 from COP dimension).

### Validity

Convergent and discriminant validity results were assessed for the 16-item version of the MusiQoL-MCAT and are shown in Table 3 . Our findings were consistent with our assumptions. Age was negatively correlated with ADL, SYMP, SSL, and REJ dimension scores. RFR dimension scores were significantly higher in women. Individuals with higher educational levels had significantly better scores, except for the SYMP, RFA, and SSL dimensions. Among single individuals, significantly lower scores were observed on the RFA, RHCS, and SSL dimensions. Unemployed people had significantly lower scores on 5 dimensions (ADL, PWB, SYMP, COP, and REJ) compared to active individuals. Disease duration was negatively correlated with ADL and REJ scores. Significant differences were observed for ADL, RHCS, and REJ dimension scores between the 4 MS subtypes, with the highest scores found in individuals with CIS and the lowest scores found in those with SP. Bonferroni pairwise post-hoc tests for the MS subtypes are presented in Appendix 1. The EDSS score was negatively correlated with all the dimensions scores of the MusiQoL-MCAT, except for the RFR and RFA dimensions. Finally, significant positive correlations were found between the MusiQoL-MCAT dimension scores and the SF-36 dimension scores. As expected, the ADL dimension of the MusiQoL-MCAT was highly correlated with the physical-like dimensions (physical function and role-physical) and the physical composite score of the SF-36 (correlation coefficients from 0.60 to 0.78). The “mental/psychological-like” dimensions of the MusiQoL-MCAT (PWB, COP, and REJ) were highly correlated with the “mental/psychological-like” dimensions (RE and MH) and the mental composite score of the SF-36 (correlation coefficients from 0.40 to 0.65). The “social-like” dimensions of the MusiQoL-MCAT (RFR, RFA, and SSL) were moderately correlated with the social functioning domain of the SF-36 (coefficients lower than 0.40).

**TABLE 3:**
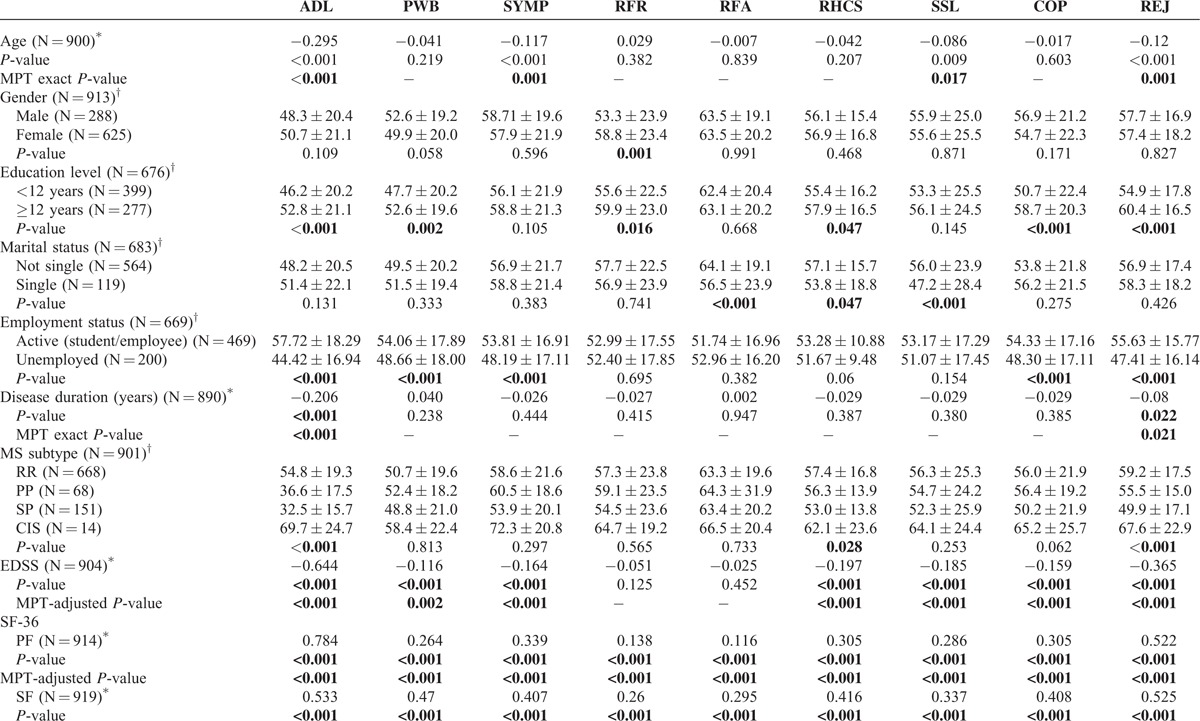
Convergent and Divergent Validity of the 16-Item MCAT Procedure

**TABLE 3 (Continued):**
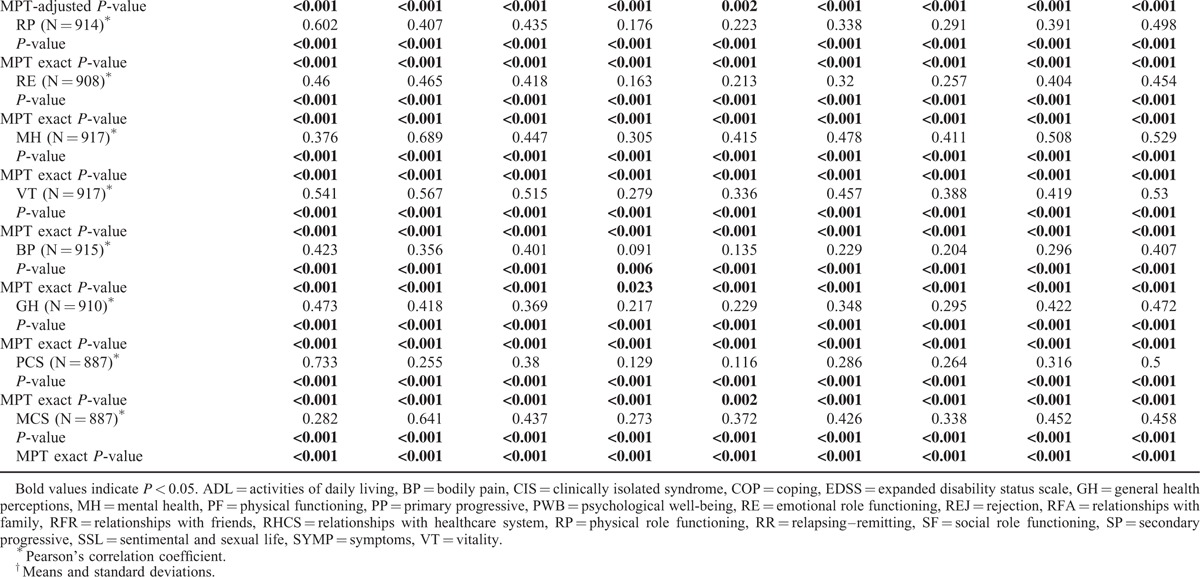
Convergent and Divergent Validity of the 16-Item MCAT Procedure

## DISCUSSION

To our knowledge, this study is one of the 1st investigations to propose a multidimensional computerized adaptive short-form questionnaire from a fixed-length available QoL questionnaire.

First, we demonstrated that the MusiQoL-MCAT had satisfactory precision and accuracy properties. All the MusiQoL-MCAT dimensions had levels of correlation higher than 0.9 with the IRT dimension scores based on the full set of items, SEM lower than 0.55 and RMSE lower than 0.3, except for 1 dimension (i.e., RHCS). However, the RHCS dimension has previously shown unsatisfactory performance, especially in the initial validation procedure.^20^ Despite this drawback, the experts and developers of the MusiQoL decided to maintain this dimension due to its specific content concerning the healthcare environment. Additionally, the external validity of the MusiQoL-MCAT was consistent with the external validity of the fixed-length MusiQoL.^20^ The MusiQoL-MCAT scores were moderately correlated with the EDSS. These results confirmed that clinical assessments may not adequately reflect patients’ perceptions and the impact of their SYMP and that the MusiQoL-MCAT adds important complementary information to traditional clinical measures. The lowest MusiQoL-MCAT scores were reported by patients with the SP form of MS, confirming that it is the most clinically aggressive and severe form of the disease. In this work, few significant differences were reported according to gender, which is consistent with other studies.^41^ Higher educational level or being in a couple was associated with higher QoL levels, as previously reported in similar cross-sectional studies.^42^ Older age was significantly associated with worse scores in the physical dimensions as ADL and SYMP, consistent with previous findings.^43^ As expected, the MusiQoL-MCAT scores were correlated with the scores of similar dimensions from the SF36-ADL dimension of the MusiQoL-MCAT with the physical-like dimensions of the SF36 and the “mental/psychological-like” dimensions of the MusiQoL-MCAT with the “mental/psychological-like” dimensions of the SF36.

From a methodological perspective, 4 key issues need to be discussed: the IRT model used; the calculation of the trait estimate after an individual gives the response; the item selection; and the stopping rule. Concerning the 1st point, 2 types of MIRT models could have been considered: between-items and within-items models.^44^ In our study, we used a between-items model (i.e., each item loading on 1 dimension only) in accordance with the steps taken previously to validate the MusiQoL.^20^ A within-item multidimensional model (i.e., 1 item loading on several dimensions) could have also been considered, but the goal of this study was not to reexamine the dimensionality of the MusiQoL. Future work should explore this option and determine whether a within-item multidimensional model better fits the data, and if it can improve the precision and accuracy properties of the MusiQoL-MCAT, especially in relationships with the healthcare dimension. Second, 2 main algorithms are available for ability estimation: ML estimation and Bayesian estimation including MAP and expected a posteriori (EAP). In our study, we used the Bayesian MAP method to estimate the latent trait level for the initial estimation of IRT scores, for updating the scores during the CAT procedure and for the final estimation of CAT scores. Although this option might be debatable, Yao^45^ has shown that MAP yielded better precision than ML and performs similarly or better than EAP. Moreover, according to Chalmers’ findings,^31^ using EAP scores for models with more than 3 factors are generally not recommended as it results in slower estimation and less precision. Therefore, MAP scores should be used instead of EAP scores for higher dimensional models,^31^ such as the MusiQoL structure. Third, the choice of the 1st item and following items is of great importance and depends on the approach taken previously (i.e., ML or Bayesian approach). In the Bayesian approach, it is recommended to select items with the highest information.^46^ For example, Petersen et al^14^ compared 2 CAT procedures, the 1st using the most informative item as the starting item and the 2nd using a less informative item and reported that administering the least or moderate informative item first provides a greater test length and a less precise measurement. Additionally, the information item selection can also be discussed. The Kullback–Liebler information item selection seemed to be the best way to select the items in our CAT procedures. Indeed, in a recent study, Yao^47^ compared the Kullback–Liebler method with 4 other methods. In many ways, the Kullback–Liebler method outperformed the other methods, producing the smallest test length, which was an important argument for clinical use of the MusiQoL. Moreover, the Kullback–Liebler information item selection is preferable to the Fisher selection, especially if the number of items used is small, as in our study.^48,49^ Fourth, we chose as a stopping criterion a fixed-length rule that was compatible with clinical practice rather than a variable-length rule which would make the questionnaire too long because of the unsatisfactory property of the relationships with healthcare dimension.

The MCAT simulation results indicated that 3 items were never administered (items 15, 16, and 25 from the SYMP and RHCS dimensions). These 3 items were the least discriminating items and provided the least amount of test information. This finding may be not surprizing because the RHCS and SYMP dimensions appear to be more influenced by a medical perspective and are further from the patient's point of view than other MusiQoL dimensions. However, other items from these 2 dimensions (i.e., items 13, 14, 23, and 24) were administered, confirming the satisfactory distribution of item exposure rates for each MusiQoL dimension. For this reason, we did not apply strategies for controlling item exposure in the MCAT.^45,50^

Last, this study also provides a broader reflection on the development strategy of the new QoL measures. Fixed-length self-reported questionnaires are classically used to measure QoL in MS and other chronic diseases. CAT has proven to be efficient compared to these classical questionnaire measurements, including increased precision and avoidance of noninformative questions. As a consequence, important groundwork has been the development of unidimensional item banks containing a large amount of items covering the entire range of a latent trait (e.g., fatigue, pain).^51,52^ The construction of a QoL item bank is an important step to proposing QoL CAT. However, a QoL item bank requires substantial resources and time because several issues remain unresolved: Is it possible to associate several QoL questionnaires based on various theoretical and conceptual backgrounds in the same bank? Can we associate generic and specific questionnaires? Should we associate questionnaires developed from the perspective of the patient and the experts? Additionally, the multidimensional nature of QoL involves the development of all of the unidimensional attributes of QoL that should be calibrated; then, the development of a multidimensional measure would be possible. All of these issues need to be resolved and therefore delay the development of a large QoL item bank and, thus, a multidimensional QoL CAT based on such a bank. Pending the completion of this major work, and although the number of items is relatively small in QoL questionnaires compared with item banks, the development of MCAT from available QoL questionnaires can be an attractive option based on financial and time resources.

### Strengths and Limitations

A limitation in our study is that we used the entire sample only for the MIRT model calibration. MCAT simulations were performed using only the complete response patterns. To overcome this issue, it should be possible to use a well-known data imputation method, such as the multiple imputations approach, and use the imputed dataset for both MIRT model calibration and MCAT simulations. Using multiple imputations on our dataset for MIRT calibration resulted in a deterioration of the model fit. This approach encouraged us to use the raw dataset in this study, given that the sample was large enough to obtain robust results.

Even with the large overall sample size in this study, the representativeness of our sample should be discussed. Compared with the most important longitudinal studies that parallel the present study, our patients were younger or older (others had mean ages of 42,^53^ 44,^54^ and 34 years),^55^ had less severe baseline disability statuses (mean EDSS scores of 4.1^53^ and 5.1^54^ were seen in other studies), and had a sex-ratio of 3:1 (4:1,^53^ 2:1,^54^ and 2.5:1^55^ were found in other studies). Future research with different sample characteristics could improve the generalizability and applicability of the MusiQoL-MCAT.

The responsiveness or sensitivity to change was not tested in our study. This property, defined as the ability to detect a meaningful change, is a core psychometric property of measurement instruments.^56^ This property is of major interest for the follow-up of patients with MS in clinical practice and for psychosocial research.^57,58^ This property should thus be confirmed on the MusiQoL-MCAT in future longitudinal studies.

Despite these limitations, our work has several strengths that should be recognized (e.g., a large sample size and psychometric properties performed in accordance with international guidelines for developing questionnaires).^14,59^ Moreover, it should be noted that these requirements are not systematically met for more “objective” outcome measures used by clinicians and decision makers.^60^ Requirements that are too high-level may cause more harm than good, especially by preventing the use and diffusion of current QoL measures. In this sense, this new multidimensional computerized adaptive short-form questionnaire has satisfactory properties and can be considered interesting option for promoting both the use and usefulness of measuring QoL in MS clinical practice.

## CONCLUSION

The MusiQoL-MCAT presents satisfactory properties and can individually tailor QoL assessment to each patient, making QoL assessment less burdensome to patients with multiple sclerosis and better adapted for use in clinical practice. As the construction of QoL item banks requires substantial resources and time, the development of MCAT from available QoL questionnaires using relevant methodology can be an attractive option based on financial and time resources.

## Supplementary Material

Supplemental Digital Content

## References

[1] Mitchell AJ Benito-León J González J-MM Quality of life and its assessment in multiple sclerosis: integrating physical and psychological components of wellbeing. *Lancet Neurol* 2005; 4:556–566. 16109362 10.1016/S1474-4422(05)70166-6

[2] Solari A Role of health-related quality of life measures in the routine care of people with multiple sclerosis. *Health Qual Life Outcomes* 2005; 3:16. 15777478 10.1186/1477-7525-3-16 PMC555749

[3] Greenhalgh J Long AF Flynn R The use of patient reported outcome measures in routine clinical practice: lack of impact or lack of theory? *Soc Sci Med* 1982; 60:833–843. 15571900 10.1016/j.socscimed.2004.06.022

[4] Morris J Perez D McNoe B The use of quality of life data in clinical practice. *Qual Life Res Int J Qual Life Asp Treat Care Rehabil* 1998; 7:85–91. 10.1023/a:1008893007068 9481154

[5] Baumstarck K Boyer L Boucekine M Measuring the quality of life in patients with multiple sclerosis in clinical practice: a necessary challenge. *Mult Scler Int* 2013; 524894. 10.1155/2013/524894 PMC3603557 23533758

[6] Walker J Böhnke JR Cerny T Development of symptom assessments utilising item response theory and computer-adaptive testing – a practical method based on a systematic review. *Crit Rev Oncol Hematol* 2010; 73:47–67. 19375939 10.1016/j.critrevonc.2009.03.007

[7] Echteld MA Deliens L Onwuteaka-Philipsen B EORTC QLQ-C15-PAL: the new standard in the assessment of health-related quality of life in advanced cancer? *Palliat Med* 2006; 20:1–2. 16482751 10.1191/0269216306pm1090ed

[8] Embretson SE Reise SP Item Response Theory for Psychologists. Mahwah, NJ: Psychology Press; 2000.

[9] Fayers P Machin D Quality of Life: The Assessment, Analysis and Interpretation of Patient-reported Outcomes. 2007; Chichester, UK: Wiley, 2nd ed.

[10] Weiss DJ Computerized adaptive testing for effective and efficient measurement in counseling and education. *Meas Eval Couns Dev* 2004; 37:70–84.

[11] Reeve BB Hays RD Bjorner JB Psychometric evaluation and calibration of health-related quality of life item banks: plans for the Patient-Reported Outcomes Measurement Information System (PROMIS). *Med Care* 2007; 45:S22–S31. 17443115 10.1097/01.mlr.0000250483.85507.04

[12] Hill CD Edwards MC Thissen D Practical issues in the application of item response theory: a demonstration using items from the pediatric quality of life inventory (PedsQL) 4.0 generic core scales. *Med Care* 2007; 45:S39–S47. 17443118 10.1097/01.mlr.0000259879.05499.eb

[13] Gardner W Kelleher KJ Pajer KA Multidimensional adaptive testing for mental health problems in primary care. *Med Care* 2002; 40:812–823. 12218771 10.1097/00005650-200209000-00010

[14] Petersen MA Groenvold M Aaronson N Multidimensional computerized adaptive testing of the EORTC QLQ-C30: basic developments and evaluations. *Qual Life Res* 2006; 15:315–329. 16547770 10.1007/s11136-005-3214-z

[15] Haley SM Ni P Dumas HM Measuring global physical health in children with cerebral palsy: illustration of a multidimensional bi-factor model and computerized adaptive testing. *Qual Life Res Int J Qual Life Asp Treat Care Rehabil* 2009; 18:359–370. 10.1007/s11136-009-9447-5 PMC2692519 19221892

[16] Dumas HM Rosen EL Haley SM Measuring physical function in children with airway support: a pilot study using computer adaptive testing. *Dev Neurorehabil* 2010; 13:95–102. 20222770 10.3109/17518420903386179

[17] Makransky G Glas CAW The applicability of multidimensional computerized adaptive testing for cognitive ability measurement in organizational assessment. *Int J Test* 2013; 13:123–139.

[18] Nikolaus S Bode C Taal E Items and dimensions for the construction of a multidimensional computerized adaptive test to measure fatigue in patients with rheumatoid arthritis. *J Clin Epidemiol* 2013; 66:1175–1183. 23958376 10.1016/j.jclinepi.2013.05.010

[19] Turner-Bowker DM Saris-Baglama RN DeRosa MA A computerized adaptive version of the SF-36 is feasible for clinic and Internet administration in adults with HIV. *AIDS Care* 2012; 24:886–896. 22348336 10.1080/09540121.2012.656573

[20] Simeoni MC Auquier P Fernandez O Validation of the Multiple Sclerosis International Quality of Life questionnaire. *Mult Scler* 2008; 14:219–230. 17942521 10.1177/1352458507080733

[21] McKenna SP Measuring quality of life in schizophrenia. *Eur Psychiatry J Assoc Eur Psychiatr* 1997; 12 Suppl 3:267s–274s. 10.1016/S0924-9338(97)89096-7 19698579

[22] McDonald WI Compston A Edan G Recommended diagnostic criteria for multiple sclerosis: guidelines from the International Panel on the diagnosis of multiple sclerosis. *Ann Neurol* 2001; 50:121–127. 11456302 10.1002/ana.1032

[23] Lublin FD Reingold SC Defining the clinical course of multiple sclerosis: results of an international survey. National Multiple Sclerosis Society (USA) Advisory Committee on Clinical Trials of New Agents in Multiple Sclerosis. *Neurology* 1996; 46:907–911. 8780061 10.1212/wnl.46.4.907

[24] Kurtzke JF Rating neurologic impairment in multiple sclerosis: an expanded disability status scale (EDSS). *Neurology* 1961; 33:1444–1452. 6685237 10.1212/wnl.33.11.1444

[25] Ware JE Snow KK Kosinski M SF-36 Health Survey: Manual and Interpretation Guide. Boston, Massachusetts: The Health Institute, New England Medical Center; 1993.

[26] Samejima F Normal ogive model on the continuous response level in the multidimensional latent space. *Psychometrika* 1974; 39:111–121.

[27] Yao L Schwarz RD A multidimensional partial credit model with associated item and test statistics: an application to mixed-format tests. *Appl Psychol Meas* 2006; 30:469–492.

[28] Rubin DB Multiple Imputation for Nonresponse in Surveys. Canada: John Wiley & Sons; 1987.

[29] Peyre H Leplège A Coste J Missing data methods for dealing with missing items in quality of life questionnaires. A comparison by simulation of personal mean score, full information maximum likelihood, multiple imputation, and hot deck techniques applied to the SF-36 in the French 2003 decennial health survey. *Qual Life Res* 2010; 20:287–300. 20882358 10.1007/s11136-010-9740-3

[30] Boyer L Baumstarck K Michel P Statistical challenges of quality of life and cancer: new avenues for future research. *Expert Rev Pharmacoecon Outcomes Res* 2014; 14:19–22. 24378121 10.1586/14737167.2014.873704

[31] Chalmers RP mirt: a multidimensional item response theory package for the R environment. *JSS J Stat Softw* 2012; 48:1–29.

[32] Cai L Metropolis-Hastings Robbins-Monro algorithm for confirmatory item factor analysis. *J Educ Behav Stat* 2010; 35:307–335.

[33] Bock RD Aitkin M Marginal maximum likelihood estimation of item parameters: application of an EM algorithm. *Psychometrika* 1981; 46:443–459.

[34] Wright BD Stone MH Best Test Design. Chicago, USA: Mesa Press; 1979.

[35] Zumbo BD A Handbook on the Theory and Methods of Differential Item Functioning (DIF): Logistic Regression Modeling as a Unitary Framework for Binary and Likert-Type (Ordinal) Item Scores. Ottawa: Directorate of Human Resources Research and Evaluation, Department of National Defense; 1999.

[36] Mulder J Linden WJ van der Linden WJ van der Glas CAW Multidimensional adaptive testing with Kullback-Leibler information item selection. *Elements of Adaptive Testing*. New York: Springer; 2009; 77–101.

[37] Harvill LM Standard error of measurement. *Educ Meas Issues Pract* 1991; 10:33–41.

[38] Choi SW Swartz RJ Comparison of CAT item selection criteria for polytomous items. *Appl Psychol Meas* 2009; 33:419–440. 20011456 10.1177/0146621608327801 PMC2791416

[39] Westfall PH Young SS Resampling-Based Multiple Testing: Examples and Methods for P-Value Adjustment. *John Wiley & Sons Canada* 1993.

[40] Yoder PJ Blackford JU Waller NG Enhancing power while controlling family-wise error: an illustration of the issues using electrocortical studies. *J Clin Exp Neuropsychol* 2004; 26:320–331. 15512923 10.1080/13803390490510040

[41] Benito-León J Morales JM Rivera-Navarro J Health-related quality of life and its relationship to cognitive and emotional functioning in multiple sclerosis patients. *Eur J Neurol Off J Eur Fed Neurol Soc* 2002; 9:497–502. 10.1046/j.1468-1331.2002.00450.x 12220381

[42] Baumstarck K Pelletier J Butzkueven H Health-related quality of life as an independent predictor of long-term disability for patients with relapsing-remitting multiple sclerosis. *Eur J Neurol Off J Eur Fed Neurol Soc* 2013; 20:907–914.e78–e79. 10.1111/ene.12087 23347258

[43] Turpin KVL Carroll LJ Cassidy JD Deterioration in the health-related quality of life of persons with multiple sclerosis: the possible warning signs. *Mult Scler Houndmills Basingstoke Engl* 2007; 13:1038–1045. 10.1177/1352458507078393 17895295

[44] Hartig J Höhler J Representation of competencies in multidimensional IRT models with within-item and between-item multidimensionality. *Z Für Psychol Psychol* 2008; 216:89–101.

[45] Yao L Multidimensional CAT item selection methods for domain scores and composite scores with item exposure control and content constraints. *J Educ Meas* 2014; 51:18–38.

[46] DO Segall Computerized Adaptive Testing. Def Manpow Data Cent U S Dep Def; Encyclopedia of Social Measurement, Academic Press USA, 1996.

[47] Yao L Comparing the performance of five multidimensional CAT selection procedures with different stopping rules. *Appl Psychol Meas* 2013; 37:3–23.

[48] Chang H-H Ying Z A global information approach to computerized adaptive testing. *Appl Psychol Meas* 1996; 20:213–229.

[49] Wang C Chang H-H Boughton KA Kullback–Leibler information and its applications in multi-dimensional adaptive testing. *Psychometrika* 2010; 76:13–39.

[50] Zheng Y Chang C-H Chang H-H Content-balancing strategy in bifactor computerized adaptive patient-reported outcome measurement. *Qual Life Res Int J Qual Life Asp Treat Care Rehabil* 2013; 22:491–499. 10.1007/s11136-012-0179-6 22538634

[51] Petersen MA Giesinger JM Holzner B Psychometric evaluation of the EORTC computerized adaptive test (CAT) fatigue item pool. *Qual Life Res Int J Qual Life Asp Treat Care Rehabil* 2013; DOI: 10. 1007/s11136-013-0372-2 (in press). 10.1007/s11136-013-0372-2 23446449

[52] Anatchkova MD Saris-Baglama RN Kosinski M Development and preliminary testing of a computerized adaptive assessment of chronic pain. *J Pain Off J Am Pain Soc* 2009; 10:932–943. 10.1016/j.jpain.2009.03.007 PMC2763618 19595636

[53] Guarnaccia JB Aslan M O’Connor TZ Quality of life for veterans with multiple sclerosis on disease-modifying agents: relationship to disability. *J Rehabil Res Dev* 2006; 43:35–44. 16847770 10.1682/jrrd.2004.12.0158

[54] Visschedijk MA Uitdehaag BM Klein M Value of health-related quality of life to predict disability course in multiple sclerosis. *Neurology* 2004; 63:2046–2050. 15596748 10.1212/01.wnl.0000145769.51420.ed

[55] Nortvedt MW Riise T Myhr KM Quality of life as a predictor for change in disability in MS. *Neurology* 2000; 55:51–54. 10891905 10.1212/wnl.55.1.51

[56] Kazis LE Anderson JJ Meenan RF Effect sizes for interpreting changes in health status. *Med Care* 1989; 27:S178–S189. 2646488 10.1097/00005650-198903001-00015

[57] Baumstarck K Butzkueven H Fernández O Responsiveness of the Multiple Sclerosis International Quality of Life questionnaire to disability change: a longitudinal study. *Health Qual Life Outcomes* 2013; 11:127. 23895207 10.1186/1477-7525-11-127 PMC3735484

[58] Michel P Auquier P Baumstarck K Development of a cross-cultural item bank for measuring quality of life related to mental health in multiple sclerosis patients. *Qual Life Res Int J Qual Life Asp Treat Care Rehabil* 2015; DOI: 10. 1007/s11136-015-0948-0 (in press). 10.1007/s11136-015-0948-0 25712324

[59] Wainer H Dorans NJ Computerized Adaptive Testing: A Primer. Mahwah, NJ, USA: Taylor & Francis Group; 2000.

[60] Boyer L Auquier P The lack of impact of quality-of-life measures in schizophrenia: a shared responsibility? *Pharmacoeconomics* 2012; 30:531–532.532–533. 22551037 10.2165/11633640-000000000-00000

