# Supplementary material for: A Multidimensional Computerized Adaptive Short-Form Quality of Life Questionnaire Developed and Validated for Multiple Sclerosis: The MusiQoL-MCAT
Source: Medicine (Baltimore). 2016 Apr 8;95(14):e3068. doi: 10.1097/MD.0000000000003068 (PMC4998748; doi:10.1097/MD.0000000000003068)
Supplement: Supplemental Digital Content [file medi-95-e3068-s001.doc]

**Appendix 1. Bonferroni pairwise post hoc tests for the MS subtypes.**

ADL: Activities of Daily Living, RHCS: Relationships with Healthcare System, REJ: Reject.

Post-hoc tests were performed only for significant differences.

|  | **ADL** | **RHCS** | **REJ** |
| --- | --- | --- | --- |
| RR (N = 668) | 54.8±19.3 | 57.4±16.8 | 59.2±17.5 |
| PP (N = 68) | 36.6±17.5 | 56.3±13.9 | 55.5±15.0 |
| SP (N = 151) | 32.5±15.7 | 53.0±13.8 | 49.9±17.1 |
| CIS (N = 14) | 69.7±24.7 | 62.1±23.6 | 67.6±22.9 |
| p-value | **<0.001** | **0.028** | **<0.001** |
| p-value RR vs PP | **<0.001** | 1.000 | 0.596 |
| p-value RR vs SP | **<0.001** | **0.019** | **<0.001** |
| p-value RR vs CIS | **0.021** | 1.000 | 0.427 |
| p-value PP vs SP | 0.807 | 1.000 | 0.172 |
| p-value PP vs CIS | **<0.001** | 1.000 | 0.106 |
| p-value SP vs CIS | **<0.001** | 0.277 | **0.002** |
